# Baicalin Exhibits a Protective Effect against Cisplatin-Induced Cytotoxic Damage in Canine Renal Tubular Epithelial Cells

**DOI:** 10.3390/metabo13121173

**Published:** 2023-11-24

**Authors:** Yao Wang, Xiao Li, Chuanguo Yan, Liuwei Xie, Yang Yang

**Affiliations:** 1 College of Police Dog Technology, Criminal Investigation Police University of China, Shenyang 110854, China; 2018990094@cipuc.edu.cn (Y.W.); 2018990026@cipuc.edu.cn (C.Y.); 2 Institute of Special Wild Economic Animals and Plants, Chinese Academy of Agricultural Sciences, Changchun 130112, China; 82101212443@caas.cn; 3 The Second Affiliated Hospital of Shenyang Medical College, Shenyang 110031, China

**Keywords:** baicalin, cisplatin, canine renal tubular epithelial cells, toxic injury

## Abstract

Renal failure is a common chronic disease in dogs that substantially affects both their quality of life and longevity. The objective of this study was to assess the protective mechanisms of baicalin in cisplatin-induced Madin–Darby canine kidney (MDCK) epithelial cells’ apoptosis model and explore the impacts of baicalin at varying doses on various indexes, such as cisplatin-induced MDCK cell apoptosis, oxidation and antioxidation, and inflammatory factors. (Methods) MDCK cells in the logarithmic growth phase were randomly divided into a control group, a model group (20 μmol/L cisplatin), and a baicalin-protection group (20 μmol/L cisplatin + 50, 25 μmol/L baicalin) and received the corresponding treatments for 24 h. The effects of cisplatin on MDCK cell apoptosis, oxidation and antioxidation, inflammatory factors, and other indicators were studied, and the relieving effect of baicalin on cisplatin-induced MDCK cell damage was explored. Calcein/PI staining and Annexin V-FITC/PI staining showed that cisplatin induced the apoptosis of MDCK cells, while baicalin effectively reduced the damage caused by cisplatin. The ELISA results demonstrated a significant elevation in the nitric oxide (NO) and malondialdehyde (MDA) levels within the MDCK cells following treatment with cisplatin (*p* < 0.01). In addition, superoxide dismutase (SOD), glutathione peroxidase (GSH), and catalase (CAT) activities remarkably declined (*p* < 0.01), while tumor necrosis factor α (TNF-α), interleukin-1β (IL-1β), and interleukin-6 (IL-6) expression within the MDCK cells were apparently elevated (*p* < 0.01). However, baicalin treatment resulted in opposite changes in these factors. The findings suggested that baicalin exhibits potential in mitigating cisplatin-induced oxidative stress and inflammation in MDCK cells. As revealed with the Western blot results, cisplatin promoted P62, P53, and BAX protein levels, increased mTOR phosphorylation, inhibited AMPK phosphorylation, and reduced Beclin1 and BCL-2 protein levels. However, a contrasting trend was observed following baicalin treatment. Cisplatin can inhibit the activity of MDCK cells, lead to abnormalities in oxidation and antioxidation functions and cell inflammatory factors, and accelerate cell apoptosis. Moreover, baicalin can significantly alleviate the damage of cisplatin to MDCK cells.

## 1. Introduction

In recent years, the growing demand for companion animals, influenced by changes in population structure and lifestyle [1], has propelled the rapid development of the pet industry [2]. Pets, particularly dogs, have assumed an important role in families due to the physiological and psychological companionship they offer. However, pet owners frequently exhibit excessive indulgence of their pets, such as providing unrestricted diets, which often elevates the risk of various chronic diseases in dogs, including kidney failure [3]. Consequently, there is an increasing focus on exploring effective strategies for preventing and curing kidney failure, improving the overall health status of dogs, and ultimately aiming to extend their lifespan.

Renal failure can lead to the rapid accumulation of metabolic waste products within the body, electrolyte imbalances, acid–base disturbances, and, eventually, a high mortality [4]. At present, kidney failure can hardly be cured and is only treated symptomatically to prolong the lifespan of patients, and such symptomatic treatment is associated with great side effects [5]. Kidney failure is accompanied by immune dysregulation, inflammatory response, and renal dysfunction in dogs, which have greatly affected the life and living conditions of dogs in some severe cases. Under the background of canine fine feeding, improving the body’s antioxidative capacity and immune function in dogs through natural drug components has been an effective way of reducing the occurrence of disease in the current canine feeding industry. However, feed supplements or veterinary medicinal products that are extracted from natural medicinal plants and can effectively improve canine immune system’s development are lacking in the industry for the time being. Therefore, it is of great significance to search for natural drug components with significant preventive or therapeutic effects and no side effects to improve the overall performance of dogs.

Cisplatin, a broad-spectrum anticancer drug, has been well-recognized to induce hepatorenal toxicity and serves as a model drug for investigating the protection on the liver and kidneys [6,7]. The cytotoxic effect of cisplatin is mainly induced by the cytotoxic effect resulting from its accumulation in the kidney. Pharmacokinetics revealed that the accumulation amount of cisplatin in the kidney is higher than that in the plasma or other tissues. When cisplatin enters the body, 90% of it can bind to plasma proteins, and it is mostly excreted directly via the kidney. Moreover, it displays the high-aggregation, high-excretion, and high-metabolism characteristics of the kidney. A large dose or continuous administration of cisplatin may induce irreversible, acute necrosis of proximal renal tubular epithelial cells, renal structural and functional disorders, and renal failure or even death in some severe cases [8]. 

Baicalin is a flavonoid compound with a relatively high content in the *Scutellaria baicalensis* Georgi, a perennial herb of the Labiaceae family. Baicalin possesses various properties such as antioxidant, anti-inflammatory, and autophagy-regulating effects [9]. It can exert protective effects on cellular toxicity. Baicalin can mitigate cellular damage, maintain cellular function and stability, and contribute to the normal functioning of tissues and organs [10]. Modern research has suggested that baicalin exhibits diverse effects, such as clearing away heat and toxic materials, anti-inflammation, antioxidation, and immunoregulation [11,12]; therefore, it has been used to treat a variety of inflammatory diseases, including periodontitis, hepatitis, and ulcerative colitis [13,14]. Moreover, baicalin also exerts a certain renal-protective effect. Zhang et al. [15] discovered that baicalin improved lead poisoning-induced oxidative injury in the kidneys of mice. Lin et al. [16] found that baicalin alleviated oxidative stress and renal ischemia–reperfusion injury to improve renal function. However, research on the role of baicalin in canine renal failure-related inflammation is rare. The role and mechanism of baicalin in ameliorating the inflammation associated with canine renal failure remain unclear. Therefore, the aim of this study was to evaluate the protective mechanisms of baicalin in cisplatin-induced Madin–Darby canine kidney (MDCK) epithelial cells’ apoptosis model. The study explored the impacts of baicalin at varying doses on various indexes, such as the cisplatin-induced MDCK cellular structure, cell apoptosis, oxidation and antioxidation, and inflammatory factors, and it assessed its effect on alleviating MDCK cell injury. The study will provide an important theoretical foundation and reference for the application of baicalin in feed as a feed additive or veterinary medicinal product.

## 2. Materials and Methods

### 2.1. Cells and Materials

This work acquired MDCK cells from the Cell Bank of the Shanghai Institutes for Biological Sciences, in the Chinese Academy of Sciences. Baicalin, a standard substance, was procured from Solarbio Biotechnology Co., Ltd. (CAS: 21967-41-9, Molecular Formula: C_21_H_18_O_11_; Beijing, China). 

### 2.2. Cell Culture

The MDCK cells were cultivated within DMEM and maintained in a 5% CO_2_ incubator under 37 °C, with regular medium change. Cells in the logarithmic growth stage and reaching 90% of confluence were collected for 0.25% trypsin digestion. The cells (approximately 9 × 10^3^/well) were inoculated into a 96-well plate for a 24 h period to allow cell adherence, followed by a 24 h treatment using cisplatin at varying doses (10–160 μmol/L). The DMEM (Cat# KGM12800-500) was purchased from KeyGEN Biotech Co., Ltd. (Nanjing, China). The trypsin (Cat# BL512B) was purchased from Biosharp Co., Ltd. (Hefei, China). The cisplatin (Cat# 61825-94-3) was purchased from Sigma-Aldrich Co. (Shanghai, China).

### 2.3. Drug Treatment

To establish the cell model, cisplatin at 20 μmol/L was added for cell treatment. Before cell adherence, baicalin at 50, 25, 20, and 15 μmol/L was added for a 12 h cell pretreatment to determine the optimal protective concentration. Each treatment was repeated six times, and the formula below was employed for calculating cell viability: cell viability = (As − Ab)/(Ac − Ab) × 100%. In this formula, As stands for the experimental well’s absorbance (including cells, culture medium, and CCK-8 reagent); Ab indicates the blank well’s absorbance (including culture medium and CCK-8 reagent), whereas Ac is the control well’s absorbance (including cells and culture medium, together with CCK-8 reagent). The CCK-8 reagent (Cat# C0038) was purchased from Shanghai Beyotime Biotechnology Co., Ltd. (Shanghai, China).

### 2.4. Apoptosis Detection

After a sequential drug treatment in the culture plate, PBS was added to rinse the cells thrice. Then, to investigate the effect of baicalin on apoptotic cell death, cells were stained with Calcein/PI staining (LIVE/DEAD^®^ Viability/Cytotoxicity Kit for mammalian cells, Molecular Probes, Eugene, OR, USA; Cat# L-3224) and fluorescence microscopy (Olympus, Japan). Moreover, the following were carried out: calxanthin AM staining of living cells with green fluorescence and propyl iodide (PI) staining of dead cells with red fluorescence. The cells at the logarithmic growth phase were digested and inoculated into a 6-well cell culture plate at 9 × 10^3^ cells/mL, followed by a 24 h treatment using 20 μmol/L of cisplatin (continued culture), with the addition of baicalin for the protective group. After collection, cells from each group were subjected to flow cytometric analysis with an Annexin V-FITC/PI staining kit (Wanlei Biotechnology Co., Ltd., Shenyang, China; Cat# WLA001b) in line with specific protocols. This examination process was completed within 1 h [17]. 

### 2.5. Oxidative and Antioxidant Function Assessment

The MDCK cells at the logarithmic growth phase were treated with cisplatin prior to their transfer to a CO_2_ incubator for an additional 24 h incubation. Baicalin (25 and 50 μmol/L) was then added to the protection group. The cells were collected after trypsin digestion, and the levels of oxidative indicators (MDA) and antioxidant indicators (SOD, GSH, and CAT) were analyzed with enzyme-linked immunosorbent assay (ELISA) kits. The absorbance values at OD_450_ were measured, and the MDA levels together with the SOD, GSH, and CAT activities were calculated [18]. The MDA (Cat# A003-1-2), SOD (Cat# A001-1-2), GSH (Cat# A006-2-1), and CAT (Cat# A007-1-1) assay kits were purchased from the Nanjing Jiancheng Bioengineering Institute (Nanjing, China).

### 2.6. Detection of Cellular Inflammatory Cytokine Levels

Cells from each group were collected using a cell scraper. Later, the TNF-α, IL-1β, IL-6, and NO levels were analyzed using the corresponding kits in line with their specific protocols, and the levels of inflammatory cytokines in each group were calculated [19]. The TNF-α (Cat# JL22455), IL-1β (Cat# JL22367), and IL-6 (Cat# JL22371) ELISA kits were purchased from Shanghai Jianglai Biotechnology Co., Ltd. (Shanghai, China). NO (Cat# A012-1-2) assay kit was purchased from Nanjing Jiancheng Bioengineering Institute (Nanjing, China).

### 2.7. Western Blot Analysis

The cellular proteins were extracted using a protein extraction kit (Beyotime Biotechnology Co., Ltd., Nanjing, China; Cat# P0033) in line with the specific protocols. The protein contents were analyzed using the BCA approach (Beyotime Biotechnology Co., Ltd., Nanjing, China; Cat# P0010). The protein samples were denatured at 100 °C for 5 min, and, later, a portion of proteins were separated through sodium dodecyl sulfate-polyacrylamide gel electrophoresis (SDS-PAGE) and transferred onto a PVDF membrane (Beyotime Biotechnology Co., Ltd., Nanjing, China; Cat# FFP71). After blocking with 5% of defatted milk, the membrane was subjected to overnight antibody incubation (1:1000, Cell Signaling Technology, Inc., Danvers, MA, USA), including anti-P62 (Cat# 5114), anti-Beclin1 (Cat# 3738), anti-AMPK (Cat# 2532), anti-P-AMPK (Cat# 2531), anti-mTOR (Cat# 2983), anti-P-mTOR (Cat# 2971), anti-BAX (Cat# 2772), anti-BCL-2 (Cat# 15071), anti-P-53 (Cat# 9282), and anti-GAPDH (1:2000, Cell Signaling Technology, Inc.; Cat# 2118), under 4 °C. On the following day, the membrane was further incubated with secondary antibodies for a 1.5 h period under ambient temperature. After washing with TBST thrice, the membrane was further incubated with secondary antibodies for 2 h. After washing, protein expression was detected using an ECL substrate (Shanghai TianNeng Technology Co., Ltd., Shanghai, China; Cat# 180-501), and the protein expression’s intensity was analyzed using the Quantity One software (version 4.6.8) [20].

### 2.8. Data Analysis

The results were represented with mean ± standard deviation. The GraphPad Prism 6.0.4 software was employed for statistical analysis [21]. The Shapiro–Wilk normality test was used for the normality test prior to the analyses. The intergroup differences were evaluated using a one-way analysis of variance (ANOVA), where *p* < 0.05 indicated significant differences among diverse treatments, while *p* < 0.01 suggested extremely significant differences.

## 3. Results

### 3.1. Modeling Concentration and Safe Concentration of Baicalin

**Figure 1:**
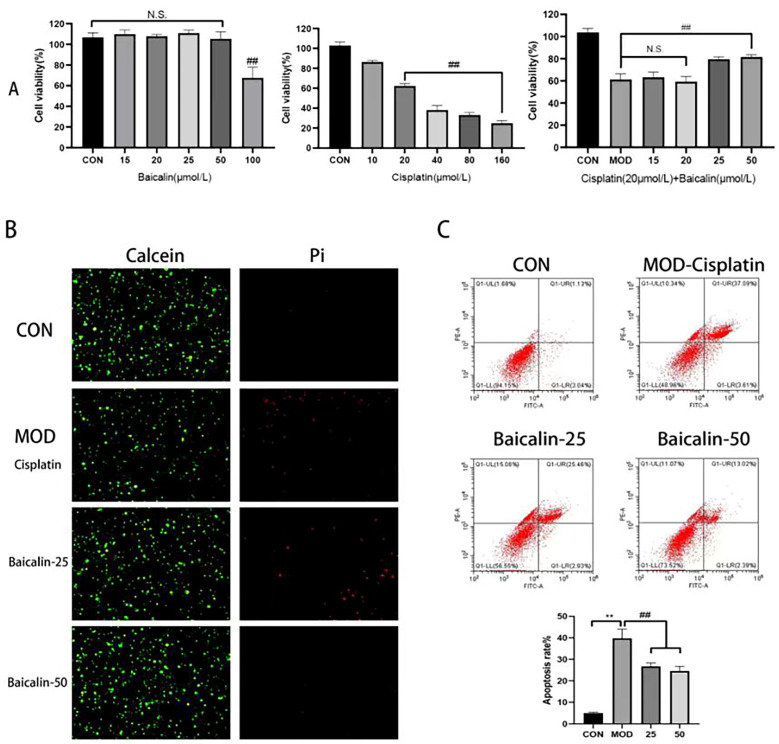
(**A**): Safe concentration of baicalin, modeling concentration of cisplatin, effective protective concentration of baicalin; (**B**): Calcein/PI staining; (**C**): Flow cytometry detection of cell apoptosis. CON: MDCK cells without treatment; MOD: cisplatin 20 μmol/L; Baicalin-25: cisplatin 20 μmol/L + baicalin 25 μmol/L; Baicalin-50: cisplatin 20 μmol/L + baicalin 50 μmol/L. N.S., no significance; ** and ##, *p* < 0.01.

To determine the safe and protective concentrations of baicalin, the MDCK cells were treated with baicalin at concentrations of 15, 20, 25, 50, and 100 μmol/L, respectively, according to the canine metabolic body weights. Compared to the CON group, at the concentration of 50 μmol/L, the cells’ proliferative ability was not affected, and the cells’ survival rate was high. At the concentration of 100 μmol/L, the cells’ proliferation was significantly affected (*p* < 0.01) (Figure 1A); therefore, 50 μmol/L was selected as the safety concentration of baicalin. In comparison, the cells treated with 20 μmol/L of cisplatin exhibited a significantly decreased cell viability relative to the CON group (*p* < 0.01), and there was little difference among the high-concentration groups as the cisplatin concentration increased, with a high cell lethality rate. Such result indicated that 20 μmol/L of cisplatin already caused a significant toxicity to cells’ growth, so 20 μmol/L of cisplatin was chosen as the modeling concentration. On this basis, baicalin at 15, 20, 25, and 50 μmol/L was added for a 12 h cell pretreatment, followed by the induction of injury with 20 μmol/L of cisplatin. Among these concentrations, the cells treated with baicalin at 25 and 50 μmol/L exhibited a significantly increased cell survival rate relative to the MOD group, and the survival rates of the cells treated with baicalin at 15 and 20 μmol/L were not significantly different from that of the MOD group, indicating the protective effect of baicalin on the cisplatin-induced MDCK cells, with the best effects being observed at 25 and 50 μmol/L (*p* < 0.01).

### 3.2. Baicalin Improved Cisplatin-Mediated MDCK Cell Apoptosis

Calcein-AM is a virtually non-fluorescent cell-permeant neutral dye that is con-verted through cell esterase into its negative, impermeant, green-fluorescent analogue [22]. Living cells are distinguished by the presence of extensive esterase activity and an intact cellular membrane. However, PI enters cells characterized by membrane degradation, a feature of late apoptosis, and cells without any membrane, a feature of necrosis (reviewed in Atale et al. [23]). The results indicated that, following cisplatin treatment, there was an increase in cells with PI staining, while cells with Calcein staining showed a decrease, suggesting that the cells underwent late apoptosis or necrosis. However, after treatment with baicalin, the number of cells with PI staining decreased, with a more significant effect observed especially at a concentration of 50 μmol/L (Figure 1B).

The cells with Annexin V-FITC/PI staining were analyzed using flow cytometry. From the flow cytometry results, compared to the CON group, the apoptosis rate of the MOD group increased by seven times, while, after baicalin intervention, the proportion of apoptotic cells remarkably decreased. Typically, the best apoptosis-inhibition effect was achieved at the concentration of 50 μmol/L, and the number of cells in the late apoptosis stage or necrotic cells apparently decreased. This outcome demonstrated a conformity with the findings obtained from the Calcein/PI staining technique. Such results suggested that cisplatin induction increased the apoptosis rate, whereas baicalin at different concentrations provided protection through reducing the apoptosis ratio of the MDCK cells, and the ability to reduce cell apoptosis was in direct proportion to the baicalin’s concentration (Figure 1C).

### 3.3. Baicalin Alleviated Cisplatin-Mediated Oxidative Stress and Inflammation of MDCK Cells

**Figure 2:**
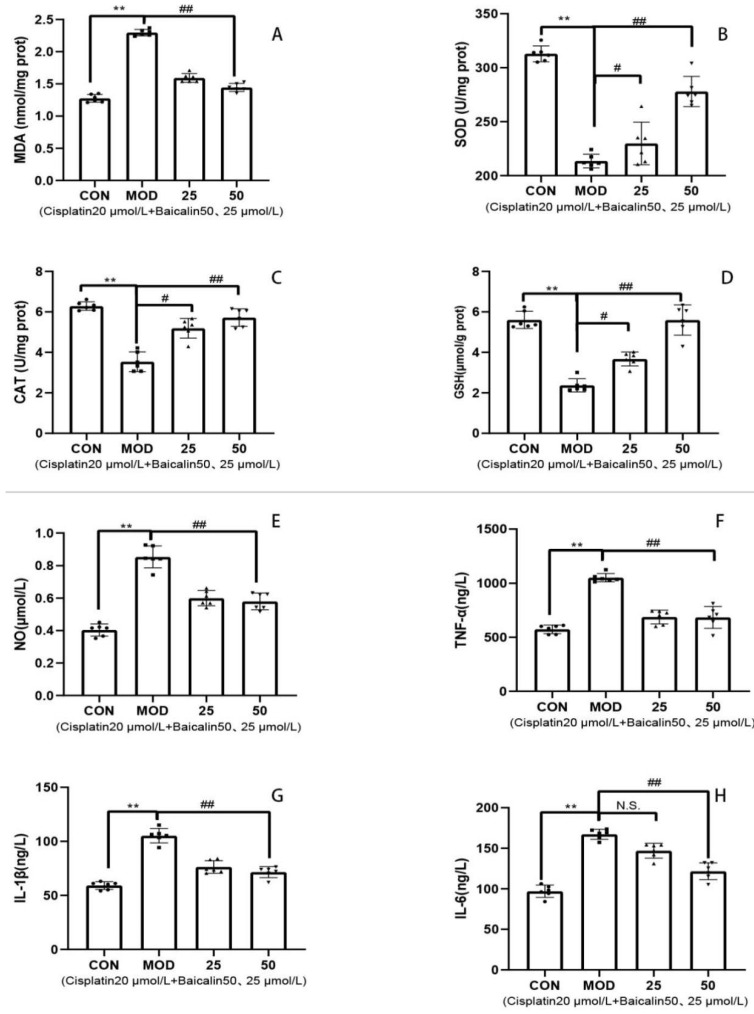
(**A**): MDA content in cells; (**B**): SOD content in cells; (**C**): CAT content in cells; (**D**): GSH content in cells; (**E**): NO content in cells; (**F**): TNF-α content in cells; (**G**): IL-1β content in cells; and (**H**): IL-6 content in cells. CON: MDCK cells without treatment. MOD: cisplatin 20 μmol/L; baicalin-25—Cisplatin 20 μmol/L + Baicalin 25 μmol/L; and baicalin-50—Cisplatin 20 μmol/L + Baicalin 50 μmol/L. N.S., no significance; #, *p* < 0.05; ** and ##, *p* < 0.01.

In a comparison with the CON group, the SOD, CAT, and GSH activities of the MOD group remarkably declined (*p* < 0.01), whereas the MDA level was evidently elevated (*p* < 0.01) (Figure 2A–D). However, relative to the MOD group, baicalin at 50 μmol/L significantly enhanced the SOD, CAT, and GSH activities (*p* < 0.01), but remarkably decreased the MDA level (*p* < 0.01). In a comparison with the CON group, the TNF-α, IL-1β, IL-6, and NO levels of the MOD group significantly increased (*p* < 0.01). However, relative to the MOD group, baicalin at 50 μmol/L significantly reduced the TNF-α, IL-1β, IL-6, and NO levels (*p* < 0.01) (Figure 2E–H). The above research results indicated that, after cisplatin induction, the MDCK cells were damaged, accompanied by an oxidative stress reaction and an inflammatory response, and that the two might be closely associated with each other. After the combined use of baicalin, the content of the oxidizing agent decreased, and the antioxidant activity was enhanced, suggesting that the oxidative stress reaction in the cells was weakened and that the oxidative toxicity was reduced. The inflammatory factor levels also dramatically decreased after baicalin application, indicating that baicalin alleviated the cisplatin-induced oxidative stress and inflammatory response in the MDCK cells.

### 3.4. Baicalin Effectively Counteracted Cisplatin-Mediated MDCK Cells Apoptosis

**Figure 3:**
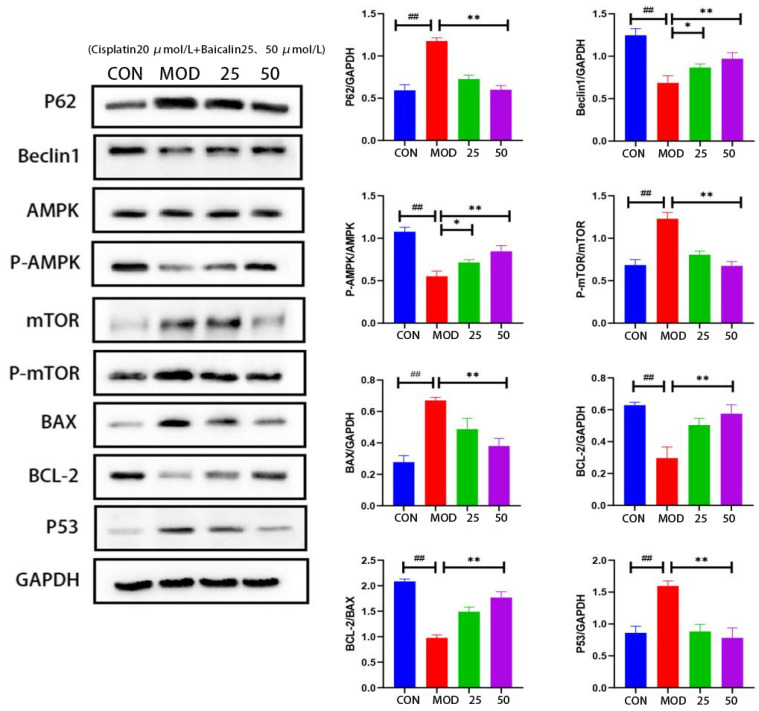
Expression levels of P62, Beclin1, AMPK, P-AMPK, mTOR, P-mTOR, BAX, BCL-2, and P53 in the cells. CON: MDCK cells without treatment. MOD: cisplatin 20 μmol/L; baicalin-25—cisplatin 20 μmol/L + baicalin 25 μmol/L; and baicalin-50—cisplatin 20 μmol/L + baicalin 50 μmol/L. *, *p* < 0.05; ** and ##, *p* < 0.01. Blue: CON group; Red: MOD group; Green: cisplatin 20 μmol/L + baicalin 25 μmol/L; Purple: isplatin 20 μmol/L + baicalin 50 μmol/L.

In comparison with the CON group, autophagy-related protein Beclin1 expression significantly decreased, while p62 expression apparently increased in the MOD group (Figure 3), indicating a decrease in the autophagic activity of the MDCK cells after cisplatin induction. In comparison to the MOD group, Beclin1 expression dramatically increased in the baicalin intervention group, while p62 expression apparently decreased, indicating that baicalin improved the autophagic function in the MDCK cells after cisplatin-induced injury.

The AMPK–mTOR axis plays an important role in energy metabolism and cell autophagy, and p53 can regulate the mTOR upstream regulatory factors to promote autophagy. Compared to the CON group, the *p*-AMPK/AMPK protein’s expression ratio decreased, whereas the *p*-mTOR/mTOR protein’s expression ratio increased in the protein samples from the MOD group, indicating a decrease in the autophagic activity. However, when baicalin was administered, the *p*-AMPK/AMPK ratio increased, while the *p*-mTOR/mTOR ratio declined, suggesting that baicalin promoted the autophagy of the MDCK cells by up-regulating *p*-AMPK or down-regulating *p*-mTOR. Additionally, compared to the CON group, the p53 and pro-apoptotic protein BAX expression apparently increased, whereas the anti-apoptotic protein BCL-2 expression remarkably decreased in the MOD group. After baicalin intervention, the p53 and BAX levels apparently decreased, while BCL-2 expression markedly increased, indicating that baicalin markedly reduced the cisplatin-induced apoptosis of the MDCK cells; the best effect was achieved at the baicalin concentration of 50 μmol/L.

## 4. Discussion

Renal failure is a common clinical disease in dogs, ranking among the top three causes of canine mortality, with a prevalence of 2–5% [24]. Although the further deterioration of the disease can be controlled in the late stage through symptomatic treatment, it significantly affects the quality of life and lifespan of the dogs [25,26]. Renal failure is often accompanied by immune dysregulation and inflammatory responses in the body [27]. Moreover, long-term antibiotic application can have adverse effects on the health of dogs. Baicalin has a low price and possesses favorable pharmacological activities related to anti-inflammation, antioxidation, and immunoregulation, with a low toxicity and minimal side effects; thus, it is a high-quality natural drug component [11]. However, research on the role of baicalin in canine renal failure-related diseases is lacking. Consequently, baicalin was selected as the object of study in this research to explore its role in mitigating MDCK cell injury.

Cisplatin is one of the first-generation platinum-based cyclin-specific antitumor drugs, exhibiting a favorable anticancer activity [28]. Despite its broad-spectrum and efficient anticancer properties, cisplatin is also associated with severe renal toxicity, making it a model drug for researching kidney protection drugs [29]. In this study, we utilized cisplatin to induce MDCK cell injury, and the results indicated that cisplatin at 20 μmol/L significantly inhibited the growth of MDCK cells, increased the apoptosis rate, induced oxidative stress and inflammation, and reduced autophagy activity in MDCK cells. It has been reported that the renal toxicity of cisplatin is primarily induced by its accumulation in the kidney, resulting in cytotoxicity [30]. Modern pharmacological research suggests that the major mechanisms of cisplatin in inducing cell death may include apoptosis, autophagy, and necrosis [31,32], which aligns with our findings. Therefore, the present results demonstrate the successful construction of a cisplatin-induced MDCK cell injury model.

This study explored the experimental concentrations of baicalin and the modeling concentrations of cisplatin based on canine metabolic body weights. Our findings revealed that cisplatin concentrations exceeding 20 μmol/L significantly inhibited MDCK cell proliferation and reduced cell survival rates. Consequently, we selected 20 μmol/L as the modeling concentration for cisplatin. Our results showed that baicalin concentrations below 50 μmol/L had no impact on the proliferation and survival rates of MDCK cells. Additionally, baicalin at 25 μmol/L and 50 μmol/L significantly promoted cisplatin-induced MDCK cell proliferation and increased survival rates. Thus, we established 50 μmol/L as the safe concentration for baicalin, with the experimental concentrations chosen as 25 μmol/L and 50 μmol/L.

This experiment utilized Calcein/PI staining and Annexin V-FITC/PI staining to assess cell apoptosis or necrosis. Our results indicated that cisplatin induction significantly promoted late apoptosis or necrosis in MDCK cells, while baicalin markedly reduced the number of late apoptotic or necrotic cells in a dose-dependent manner. The mitochondrial pathway is the classical cell apoptotic pathway, where the anti-apoptotic protein Bcl-2 and the pro-apoptotic protein BAX regulate mitochondrial structural and functional stability [33]. The results showed that, after cisplatin intervention, the BCL-2 expression level in the MDCK cells decreased, and the Bax expression level increased, indicating that cisplatin increased the apoptosis of the MDCK cells. In contrast, baicalin treatment led to opposite results in a dose-dependent manner. Thus, baicalin can mitigate cisplatin-induced MDCK cell apoptosis by regulating the mitochondrial pathway. An animal experiment revealed that *Scutellaria baicalensis* Georgi extract can ameliorate mice renal tubular cell necrosis and sloughing induced by cisplatin, with baicalin being identified as the main active component [34]. Baicalin has also been demonstrated to exert a protective effect against apoptosis in renal cells induced by various chemicals such as lead, H_2_O_2_, and methylglyoxal, and this protective effect is associated with baicalin’s reduction of the BAX/BCL2 ratio, activation of the Nrf2 pathway, and prevention of the mitochondrial membrane’s potential loss [15,35,36]. These findings suggest that baicalin can improve renal cell apoptosis induced by different chemicals through various molecular pathways.

The organism has developed a comprehensive antioxidant system. Excessive production of MDA in cells is a sign of oxidative damage, leading to an imbalance in cellular oxidation [37]. GSH consumption indicates the presence of excessive free radicals or other oxidative toxic factors in the body [38]. The levels of SOD, CAT, and GSH also reflect the metabolism of oxygen free radicals and the body’s antioxidant ability [39]. Studies conducted by Hu et al. [11] and Jia et al. [40] revealed that supplementing baicalin in a diet enhances the activities of CAT and SOD while reducing MDA content in pre-weaned calves and tilapia. Baicalin also plays an important regulatory role in ameliorating cells’ oxidative stress induced by various factors [41,42,43]. Moreover, the antioxidant activity of baicalin has been demonstrated to be mediated through the Nrf2 signaling pathway [44,45]. Our study obtained similar results; the results revealed that cisplatin treatment for 24 h significantly increased the production of MDA in the MDCK cells and decreased the activities of SOD, CAT, and GSH, while baicalin treatment significantly reversed these adverse effects in a dose-dependent manner. This further indicates that baicalin displays apparent pharmacological characteristics and animal universality in enhancing the body’s antioxidant capacity. Inflammatory responses are triggered when cells and tissues are damaged. Inflammatory cytokines have essential effects on immunoregulation and other processes [46]. The changes in inflammatory factors reflect the severity of inflammation in the body and the immune response to inflammation [47]. In this study, TNF-α, IL-6, IL-1β, and NO were selected as the indexes for evaluating inflammation. According to our findings, cisplatin treatment significantly increased the TNF-α, IL-6, IL-1β, and NO contents in the MDCK cells, indicating the occurrence of cellular inflammatory injury. Baicalin effectively repaired the inflammatory damage caused through cisplatin by reducing inflammatory factor levels, and these results were similar to those of previous research [48,49]. This suggests that baicalin, in ameliorating cisplatin-induced MDCK cell injury, not only improves oxidative stress but also exerts immunomodulatory effects. 

In mammalian cells, ATP homeostasis is closely related to cell growth, proliferation, autophagy, and apoptosis [50]. Adenosine monophosphoprotein kinase (AMPK) is a key regulatory factor for ATP homeostasis, extensively distributed in metabolic organs throughout the body, and plans an important role in maintaining energy, metabolism, and cell apoptosis [51,52]. Cell autophagy is a tightly regulated process where the cancer suppressor gene p53 can up-regulate the mammalian target of rapamycin (mTOR)’s upstream regulatory factors to promote autophagy through the transcription-dependent pathway [53]. mTOR regulates cell autophagy, while p62 is the autophagic substrate, and the disruption of cell apoptosis leads to the accumulation of p62. Consequently, p62, p53, and Beclin1 protein levels are indicative of autophagic activity [54]. Our research found that cisplatin treatment reduced Beclin1 expression but elevated p62 and p53 expression in the MDCK cells, suggesting a decrease in autophagic function. However, baicalin intervention showed the opposite effect, indicating that baicalin improved autophagic function in the MDCK cells. Additionally, the AMPK/mTOR pathway has been extensively found to be involved in autophagy regulation [55,56]. Cisplatin intervention enhanced mTOR phosphorylation and reduced AMPK phosphorylation, indicating a decreased autophagic activity. Baicalin reversed these changes, suggesting its ability to attenuate autophagy in cisplatin-induced MDCK cells by regulating the AMPK/mTOR pathway. 

Based on the current results, cisplatin-induced MDCK cell injury involves oxidative stress, inflammatory response, autophagy, and apoptosis. Baicalin, in turn, regulates and mitigates cisplatin-induced MDCK cell injury through multiple pathways and cellular physiological processes. This cellular level study will provide a reference for future research on baicalin as a feed additive in the daily diet of dogs. Overall, baicalin shows promise as a therapeutic agent for alleviating the toxic side effects associated with cisplatin treatment. However, further research is required to elucidate the underlying molecular mechanisms and assess the efficacy of baicalin in vivo. This research holds significant importance in the development of a natural drug to prevent and improve canine renal failure. 

## Data Availability

The data presented in this study are available on request from the corresponding author. The data are not publicly available due to privacy.
